# Validity of Italian administrative healthcare data in describing the real-world utilization of infusive antineoplastic drugs: the study case of rituximab use in patients treated at the University Hospital of Siena for onco-haematological indications

**DOI:** 10.3389/fonc.2023.1059109

**Published:** 2023-05-31

**Authors:** Claudia Bartolini, Giuseppe Roberto, Anna Girardi, Valentino Moscatelli, Andrea Spini, Alessandro Barchielli, Monica Bocchia, Alberto Fabbri, Sandra Donnini, Marina Ziche, Maria Cristina Monti, Rosa Gini

**Affiliations:** ^1^ Pharmaecoepidemiology Unit, Agenzia Regionale di Sanità della Toscana, Firenze, Italy; ^2^ Department of Life Sciences, Università Degli Studi di Siena, Siena, Italy; ^3^ Tuscany Cancer Registry, Istituto per lo Studio e la Prevenzione Oncologica, Firenze, Italy; ^4^ Onco-hematology Unit, Azienda Ospedaliera Universitaria Senese, Siena, Italy; ^5^ Università di Pavia, Dipartimento di Sanità Pubblica, Medicina Sperimentale e Forense, Pavia, Italy

**Keywords:** rituximab, infusive antineoplastics, validation, administrative data, drug utilisation

## Abstract

**Introduction:**

Italian administrative healthcare databases are frequently used for studies on real-world drug utilization. However, there is currently a lack of evidence on the accuracy of administrative data in describing the use of infusive antineoplastics. In this study, we used rituximab as a case study to investigate the validity of the regional administrative healthcare database of Tuscany (RAD) in describing the utilization of infusive antineoplastics.

**Methods:**

We identified patients aged 18 years or older who had received ≥1 rituximab administration between 2011 and 2014 in the onco-haematology ward of the University Hospital of Siena. We retrieved this information from the Hospital Pharmacy Database (HPD-UHS) and linked the person-level information to RAD. Patients who had received ≥1dispensing of rituximab, single administration episodes, and patients treated for non-Hodgkin Lymphoma (nHL) or Chronic Lymphocytic Leukemia (CLL) were identified in RAD and validated using HPD-UHS as the reference standard. We identified the indications of use using algorithms based on diagnostic codes (ICD9CM codes, nHL=200*, 202*; CLL=204.1). We tested 22 algorithms of different complexity for each indication of use and calculated sensitivity and positive predictive value (PPV), with 95% confidence intervals (95%CI), as measures of validity.

**Results:**

According to HPD-UHS, 307 patients received rituximab for nHL (N=174), CLL (N=21), or other unspecified indications (N=112) in the onco-haematology ward of the University Hospital of Siena. We identified 295 rituximab users in RAD (sensitivity=96.1%), but PPV could not be assessed due to missing information in RAD on dispensing hospital wards. We identified individual rituximab administration episodes with sensitivity=78.6% [95%CI: 76.4-80.6] and PPV=87.6% [95%CI: 86.1-89.2]. Sensitivity of algorithms tested for identifying nHL and CLL ranged from 87.7% to 91.9% for nHL and from 52.4% to 82.7% for CLL. PPV ranged from 64.7% to 66.1% for nHL and from 32.4% to 37.5% for CLL.

**Discussion:**

Our findings suggest that RAD is a very sensitive source of information for identifying patients who received rituximab for onco-haematological indications. Single administration episodes were identified with good-to-high accuracy. Patients receiving rituximab for nHL were identified with high sensitivity and acceptable PPV, while the validity for CLL was suboptimal.

## Introduction

In Italy, the National Health Service (NHS) provides universal healthcare assistance to all inhabitants. Healthcare services are delivered at the regional level to any subject registered with a general practitioner (1). Administrative information, based on the type of service provided (e.g., hospitalization, drug dispensing, diagnostic tests or procedures), is recorded in specific electronic databank. For these reasons, Italian administrative databases are region-wide, population-based data sources that can link patient-level information from several administrative databanks (1). Such data sources have been widely used to execute real-world pharmacoepidemiological studies in large patient populations. However, when studying infusive antineoplastics, which are used for multiple indications and administered during both ambulatory and inpatient care, concerns about data validity may arise (i.e. are data useful to accurately describe the real-world utilization of the study drug)? (2–4). This is because Italian administrative healthcare databases do not record information on the indications of use for which drugs are dispensed, and drugs administered during inpatient care are usually tracked at the hospital ward-level rather than the person-level (5). These limitations can introduce significant biases in the results of pharmacoepidemiological studies. Therefore, understanding the validity of Italian administrative healthcare data sources in studying infusive antineoplastics utilization is essential for generating solid real-world evidence from large populations of patients using these medications. However, to the best of our knowledge, no previous study has attempted to estimate the validity of Italian administrative healthcare databases in describing the utilization of any infusive antineoplastic medication.

In this regard, other existing intra-hospital electronic databases, such as those supporting the management of hospital pharmacies, could be linked to administrative healthcare databases to serve as a reference standard for obtaining estimates of validity. In a previous study (6), we linked person-level information from the Hospital Pharmacy Database of the University Hospital of Siena (HPD-UHS) to the regional administrative healthcare databases of Tuscany (RAD), Italy, and demonstrate that HPD-UHS could provide consistent information on the utilization of the infusive antineoplastic drug rituximab, including indications of use and occurrence of administration episodes during both inpatient and outpatient care. Therefore, based on our prior experience (6), in the present study, we used rituximab as the case study and the Hospital Pharmacy Database of the University Hospital of Siena as the reference standard to provide evidence on the validity of Italian administrative healthcare data in describing the real-world utilization of infusive antineoplastics.

## Materials and methods

### Data sources

In our study, we utilized two distinct data sources - the regional administrative database of Tuscany (RAD) and the Hospital Pharmacy Database of the University Hospital of Siena (HPD-UHS). Tuscany is a central Italian region with a population of approximately 3.7 million inhabitants, which constitutes about 6% of the Italian population. Siena, on the other hand, is the city where one of the largest University Hospitals of Tuscany is located.

### Regional administrative database of Tuscany

The RAD database gathers patient-level information regarding all healthcare services that are reimbursed by the NHS and provided to any person registered with a general practitioner in Tuscany (5, 7, 8). Registration with a general practitioner grants individuals the right to receive public healthcare assistance across any Italian region. The region where the person is registered with the general practitioner is responsible for reimbursing any public healthcare service delivered to him/her on the Italian territory. Patient-level information is recorded in various databanks, which can be linked by utilizing a universal pseudo-anonymized identification code. For the purposes of our study, we utilized the following RAD databanks: (1) the population registry, which we used to retrieve demographic characteristics and define the available time of observation (e.g. vital status, start/end of regional healthcare service enrollment); (2) the outpatient drug dispensing registry, which collects information on drugs dispensed to non-hospitalized patients; (3) the exemptions from co-payment registry (EXE), which records diagnoses representing the reason for exemptions from healthcare service co-payment; (4) the Emergency room admission registry (ER), and (5) Hospital discharge records (HDR), which we used to retrieve diagnoses recorded during emergency room or hospital admissions.

### The database of the Hospital Pharmacy of the University Hospital of Siena

The HPD-UHS database collects patient-level information on infusive drugs that are prepared by the hospital pharmacy. Drugs that are recorded in the database may be administered within the hospital facilities to either inpatients or outpatients. The database collects information on patients’ demographics, drug-related information (e.g., trade name, active substance, route of administration, indication of use, date of administration, treatment line, dose), and the actual hospital ward where the drug is delivered and administered to the patient.

### Dataset creation and selection of the study population

**Figure 1:**
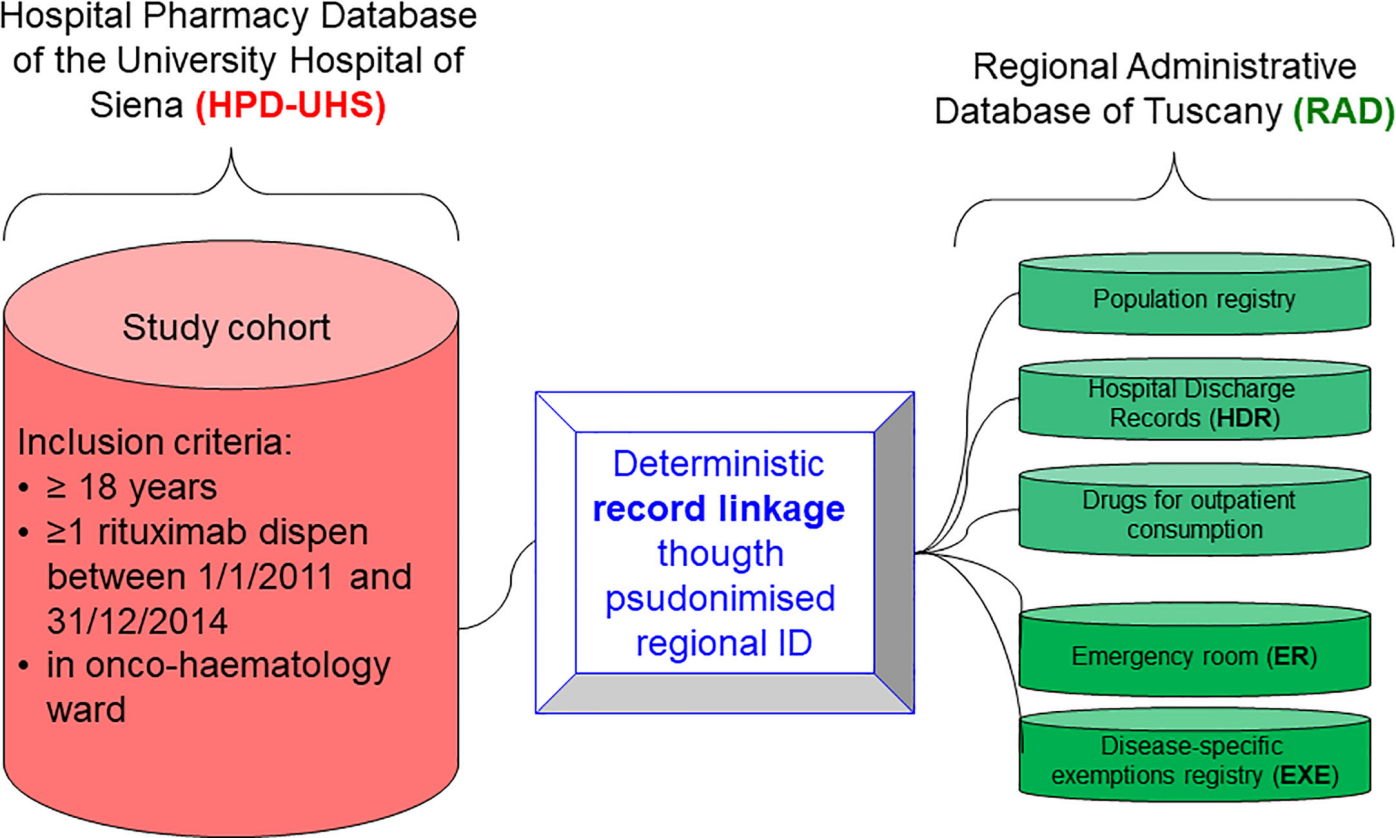
Dataset creation.

We included all patients aged 18 years and above who received rituximab treatment in the oncology or haematology wards of the University Hospital of Siena between January 1, 2011, and December 31, 2014. The competent regional authority (ESTAR - *Ente di Supporto Tecnico-Amministrativo Regionale*) performed data anonymization, and each patient was assigned a universal pseudo-anonymized identification code to allow the deterministic linkage of patient-level data contained in HPD-UHS to those collected in RAD (3) (**Figure 1**). Patients registered with the Regional Healthcare Service of an Italian region other than Tuscany were excluded from the study cohort since linkage to RAD was not possible. For each patient in the study population, we considered the date of the first recorded administration of rituximab in HPD-UHS as the cohort entry date.

### Infusive antineoplastic drug used as the study case: rituximab

Rituximab is a monoclonal antibody that is administered through intravenous infusion in hospital settings only, during both inpatient and ambulatory care. It is approved for the treatment of several conditions, including non-Hodgkin’s lymphoma (nHL), chronic lymphocytic leukemia (CLL), rheumatoid arthritis, and autoimmune vasculopathies (9). It is also used for different off-label indications in various autoimmune diseases and as a post-transplant therapy (10–14). In the case of CLL, rituximab in association with chemotherapy is indicated for the treatment of patients with previously untreated and relapsed/refractory disease. For adult patients with nHL, rituximab is indicated either as monotherapy (patients with stage III-IV follicular lymphoma who are chemo-resistant or are in their second or subsequent relapse after chemotherapy) and in association with chemotherapy (previously untreated patients with stage III-IV follicular lymphoma; patients with CD20 positive diffuse large B cell non-Hodgkin’s lymphoma). Additionally, rituximab maintenance therapy is indicated in adult follicular lymphoma patients responding to induction therapy (15).

### Validation of RAD

To validate the accuracy of RAD data in describing the real-world use of rituximab in the study population, sensitivity and positive predictive value (PPV) were calculated using HPD-UHS as the reference standard (16). Three different events were considered for the validation: 1) patients treated with rituximab, 2) single rituximab administration episodes, and 3) onco-haematological indications for rituximab administration. Therefore, assuming that the events of interest identified in HPD-UHS represent all the true events of interest in the study population, the sensitivity was calculated as the probability that all true events of interest in the study population were identified in RAD (i.e., the number of true events of interest found in RAD divided by the total number of true events of interest in the study population). The PPV was calculated as the probability that all the events of interest found in RAD were true events of interest (i.e., the number of true events of interest found in RAD divided by the total number of events found in RAD). Given that the study population was entirely represented by rituximab users according to HPD-UHS data, patients treated with rituximab according to RAD data were defined as those with at least one rituximab dispensing recorded in RAD during one year following cohort entry date.

To assess the validity of RAD in identifying single episodes of treatment administration, the study population was restricted to patients with at least one rituximab administration recorded in both RAD and HPD-UHS. Episodes of rituximab administration recorded in both RAD and HPD-UHS within three days of each other were considered the same treatment episode. The validity of RAD in identifying the indication for rituximab use was assessed using the indication of use for the first recorded rituximab administration observed in HPD-UHS as the reference standard. This analysis was limited to patients who had received at least one rituximab administration in both RAD and in HPD-UHS, and whose HPD-UHS records indicated the use of rituximab for the treatment of nHL or CLL. Thirteen simple algorithms were tested on RAD (**Table 1**) to identify patients receiving rituximab for nHL and for CLL indication using ICD9CM diagnostic codes (200* and 202* for nHL and 204.1 for CCL). These simple algorithms were characterized by a pre-specified time window during which the diagnostic codes were searched and specific administrative databanks from which codes were retrieved. In particular, the following time windows were tested: a) *ever*, any time before and after cohort entry, b) *pre*, any time before cohort entry, c) *pre2*, during the 2-year period before cohort entry, d) *within*, during hospitalization. Administrative databanks used for diagnostic codes retrieval were hospital discharge records (HDR), emergency room (ER), reasons for exemption from copayment (EXE).

**Table 1:** Description of simple algorithms used to identify in the regional administrative database of Tuscany patients treated with rituximab for nHL and CLL, respectively.

Algorithm	Databank	Pattern of records*	ICD9CM codes	
			nHL	CCL
HDR*ever*	HDR	≥1 any time before/after index date	200.xx; 202.xx	204.1
HDR*pre*	HDR	≥1 any time before index date		
HDR*pre2*	HDR	≥1 during two years before index date		
HDR*within*	HDR	≥1 during hospitalization (from admission to discharge)		
HDR*post*	HDR	≥1 any time after index date		
ER*ever*	ER	≥1 any time before/after index date		
ER*pre*	ER	≥1 any time before index date		
ER*pre*	ER	≥1 any time after index date		
EXE*ever*	EXE	≥1 any time before/after index date		
EXE*pre*	EXE	≥1 any time before index date		
EXE*pre*	EXE	≥1 any time after index date		

nHL, non-Hodgkin Lymphoma; CLL, Chronic Lymphocytic Leukemya; HDR, hospital discharge records; ER, Emergency room admissions; EXE, disease-specific exemptions from co-payement.

The sensitivity and PPV of composite algorithms, which were combinations of simple algorithms, were also calculated. **Table 1** provides a detailed description of the simple algorithms used to identify the relevant indication of use, and **Table 2** describes the composite algorithms used, twenty-two in total. Sensitivity and PPV were reported as percentages with corresponding 95% confidence intervals calculated using the Wald method (17). All analyses were executed using the statistical software Stata14 (18).

**Table 2:** Description of composite algorithms definition.

Composite algorithm	Simple algorithm combinations
#1	HDR*ever* or EXE*ever* or ER*ever*
#2	(HDR*pre* and HDR*within*) or EXE*pre* or ER*pre*
#3	HDR*pre* or HDR*within* or EXE*pre*
#4	(HDR *pre2* and HDR*within*) or EXE*pre*
#5	HDR*pre2* or EXE*pre*

HDR, hospital discharge records; ER, Emergency room admission registry; EXE, exemptions registry.

The study was approved by the Ethical Committees of the Local Health Authority “Area Vasta Sud-Est” in July 2016 (code identifier: ARSAOUS2016).

## Results

The study cohort included 307 patients who had at least one recorded rituximab administration in HPD-UHS and a valid regional anonymized identification code that was successfully linked to the population registry of RAD. According to data from HPD-UHS, 174 patients received rituximab for nHL, 21 for CLL, and 112 for other indications. Among the latter group, 98 patients were categorized as “not specified,” and the remaining 13 subjects had missing information.

RAD identified 295 of the 307 patients who were treated with rituximab in the onco-haematology ward of the University Hospital of Siena, according to HPD-UHS (sensitivity= 96.1%). However, due to missing information in RAD regarding the hospital ward where rituximab was dispensed, those patients treated with rituximab during the study period in the onco-haematology ward of the University Hospital of Siena could not identified in RAD. Therefore, PPV for identifying patients treated with rituximab could not be determined.

Regarding the identification of single episodes of rituximab administration, among the 295 patients with at least one rituximab record in both data sources, RAD had a sensitivity of 78.6% [95%CI: 76.4-80.6] and a PPV of 87.6% [95%CI: 86.1-89.2]. A total of 1,758 records of rituximab administration were found in HPD-UHS, of which 1,382 matched a rituximab dispensing recorded in RAD within ±3 days. Of the 376 treatment episodes recorded in HPD-UHS with no correspondence in RAD, 0.8% and 41.3% occurred during day hospital and ordinary hospital admissions, respectively.

**Figure 2:**
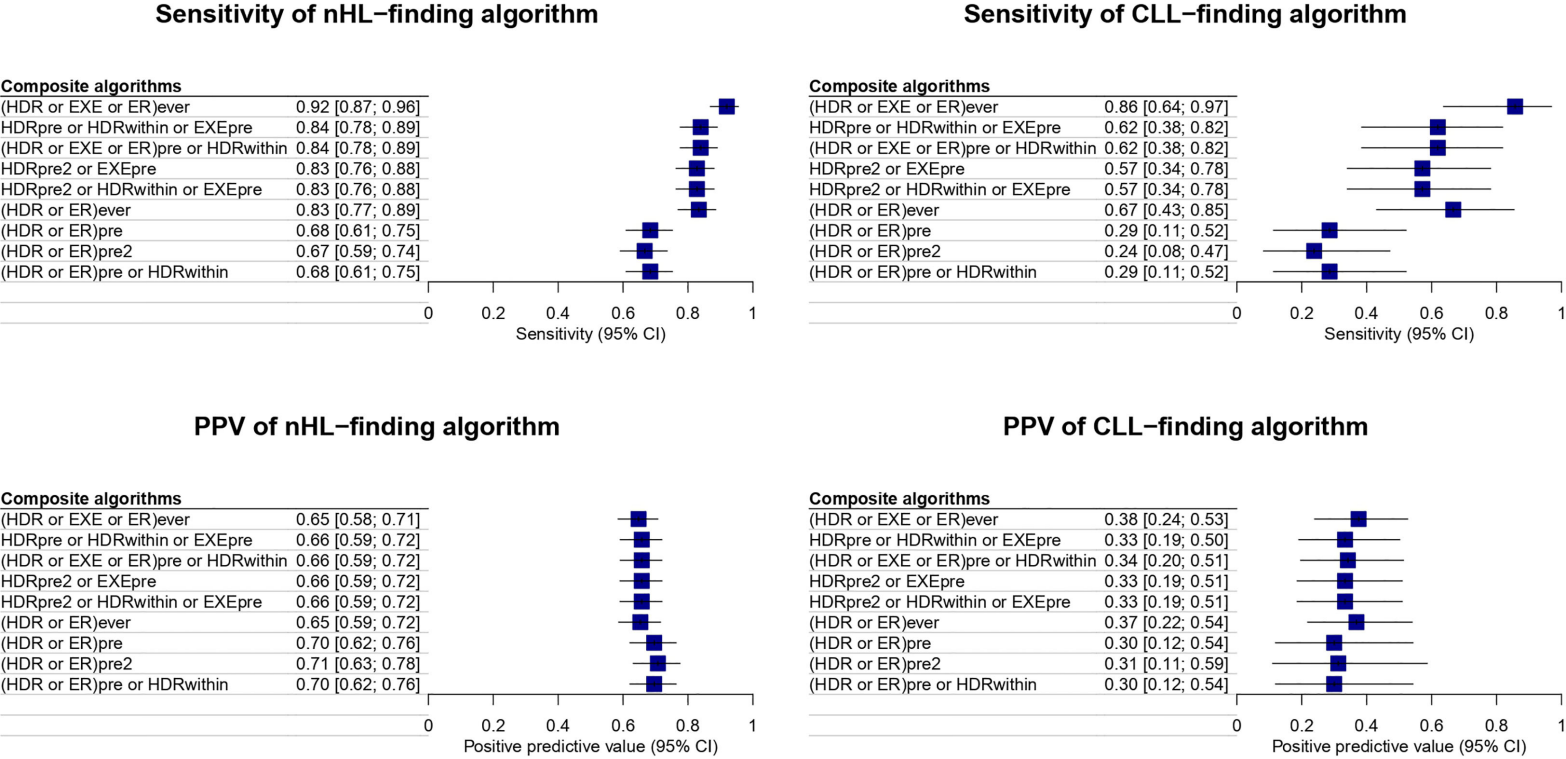
Sensitivity and PPV of simple algorithms for identifying patients treated with rituximab for CLL and nHL.

Among the 295 rituximab users identified in both data sources, 166 patients received rituximab for nHL, and 18 for CLL, according to information recorded in HPD-UHS. The sensitivity and PPV of simple algorithms for identifying patients treated with rituximab for CLL and nHL, respectively, are reported in **Figure 2** (see also **Supplementary Table 1**). The highest sensitivity for nHL was achieved by the simple algorithm HDR*ever*, resulting in a sensitivity of 83.3% [95%CI: 77.7-88.8] and a PPV of 65.3% [95%CI: 59.1-71.6]. However, when the same simple algorithm was used to identify CLL patients, the sensitivity was 57.1% [95%CI: 49.8-64.5], and the PPV was 34.3% [95%CI: 18.5-50.0]. The simple algorithms ER*pre* and ER*pre2* showed the same values of sensitivity and PPV in identifying nHL. These algorithms yielded the highest PPV, i.e., 90.1% [95%CI: 73.9-100.0], but also the lowest sensitivity of all the simple algorithms tested, i.e., 5.7% [95%CI: 2.2-9.2]. The algorithm EXE*ever* identified patients receiving rituximab for CLL with the highest value of sensitivity, i.e., 66.6% [95%CI: 59.6-73.7], and a PPV of 41.1% [95%CI: 24.6-57.7]. The simple algorithm ER*ever* showed the highest PPV among the simple algorithms for CLL identification, i.e., 75.0% [95%CI: 32.0-100.0], and a sensitivity of 14.3% [95%CI: 9.1-19.5].

**Figure 3:**
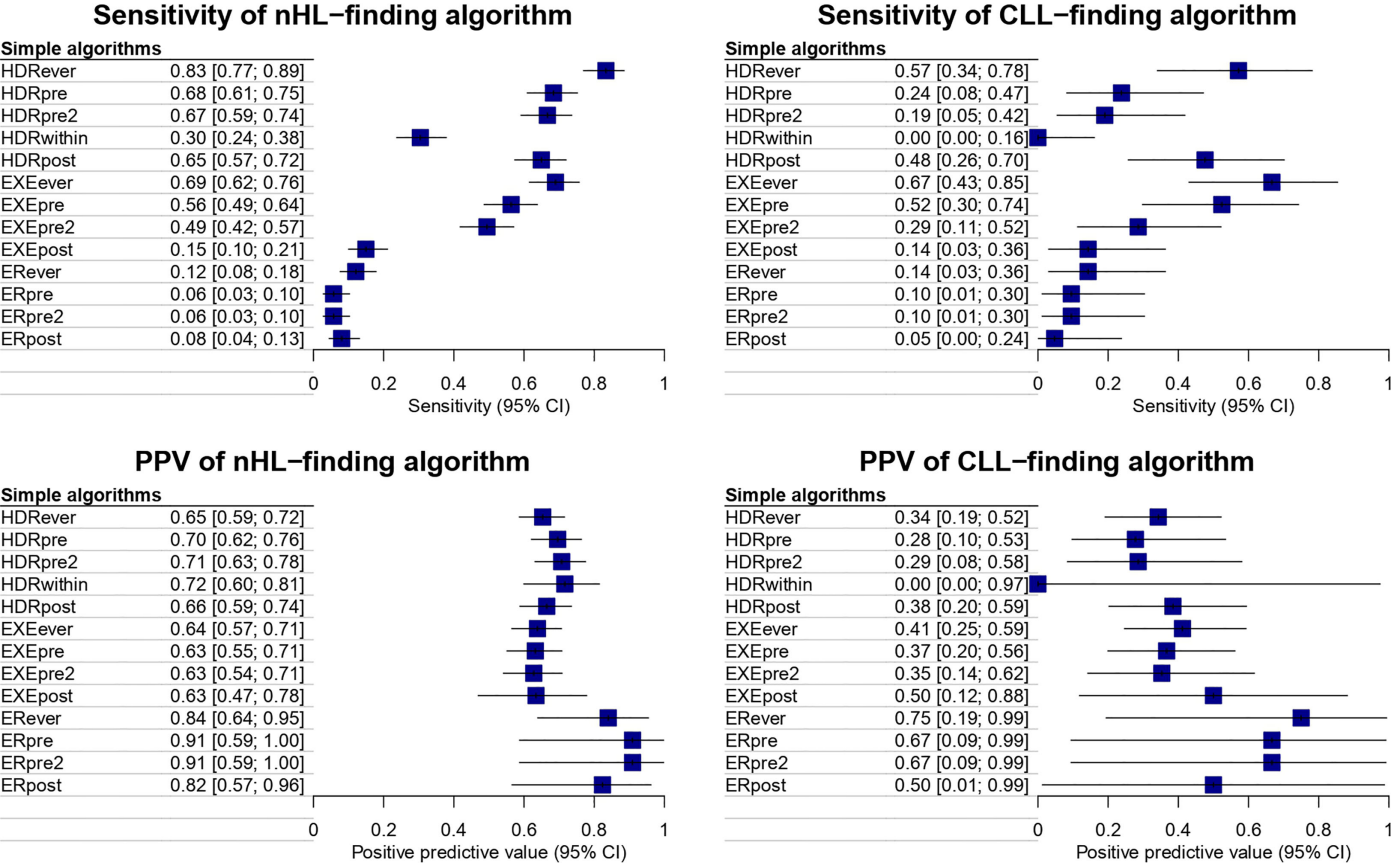
Sensitivity and PPV of complex algorithms for identifying patients treated with rituximab for CLL and nHL.

The sensitivity and PPV of complex algorithms for identifying patients treated with rituximab for CLL and nHL, respectively, are reported in **Figure 3** (see also **Supplementary Table 2**). The highest sensitivity for both nHL and CLL was achieved by the composite algorithm (HDR or EXE or ER)*ever* i.e., of 91.9% [95%CI: 87.9-95.9] and 85.7% [95%CI: 80.5-90.9], respectively. In particular, the composite algorithm (HDR or ER or EXE) *ever* also showed the highest PPV for CLL identification, i.e. 37.5% [95%CI: 23.8-52.5], The composite algorithm (HDR or ER)*pre* showed the highest PPV for nHL identification, i.e. 70.2% [95%CI: 61.7-75.7], and a sensitivity of 67.7% [95%CI:60.5-75.0].

## Discussion

The present study provides valuable evidence for researchers using Italian administrative databases to investigate the real-world utilization of infusive antineoplastics, specifically rituximab.

Findings from this study showed that the administrative healthcare database of Tuscany is a highly sensitive source of data for identifying patients treated with rituximab. RAD also identified episodes of rituximab administration with good sensitivity and high PPV. Overall, patients receiving rituximab for nHL were identified with good sensitivity and acceptable PPV while validity of algorithms tested for identifying CLL patients resulted to be suboptimal mainly due to low PPV.

The study also evaluated the reliability of using the database to identify single treatment episodes of rituximab and found that less than 22% of recorded rituximab administrations in the hospital database were not found in the administrative database. Approximately half of the administrations that were not found in the administrative database occurred during hospitalization, likely due to the administrative nature of the data recording process (i.e., information on drugs administered during inpatient care is recorded at the hospital ward-level instead of patient-level). There are two potential explanations for the remaining missing treatment episodes. The first possibility is that these episodes do not correspond to treatments that are tracked at the patient-level in RAD, because they fall outside of the approved treatment schedule or posology for reimbursement. The second possibility is that in some cases, the date of rituximab dispensing may have been incorrectly recorded outside of the allowed ±3 days with respect to the administration date recorded in HPD-UHS. This could be due to some unknown error, as can happen with real-world data. Both possibilities emphasize the importance of ensuring the accuracy and completeness of administrative healthcare data in order to provide reliable information on the real-world utilization of rituximab and other infusive antineoplastic drugs.

In this study we also provide evidence on the validity of different simple and composite algorithms for the identification of patients receiving rituximab for nHL or CLL. Overall, the algorithms that identified nHL patients resulted in higher PPVs compared to those that identified CLL patients. This is mainly due to the lower prevalence of CLL in the study population, as well as the lower granularity of CLL diagnosis codes recorded in the exemption from copayment registry. In fact, this registry only allows for the first three digits of the ICD9CM codification to be recorded, which may not provide enough detail to accurately capture cases of CLL.

It is important to note that the PPV is dependent on disease prevalence (19). For example, in a hypothetical population where all patients have CLL (prevalence=100%), the PPV of a case-finding algorithm (i.e., true cases/total cases identified by the algorithm) would always be 100% whenever at least one case is identified. Therefore, the PPV of the algorithms tested in this study may vary when applied to populations with different disease prevalence.

Overall, this study showed that regardless of the indication of interest, diagnoses recorded in the hospital discharge records or the databank of exemption from copayment registry had high values of sensitivity. On the other hand, diagnoses recorded in the emergency room admission registry had lower sensitivity compared to hospital discharge records or exemption from copayment registry, but a higher PPV. Therefore, future studies can use the findings from this work to design the most appropriate study-specific algorithm. The simple algorithms tested in this study can be combined in different ways to achieve the most suitable balance between sensitivity and PPV for the specific study objectives. The composite algorithm that performed the best in identifying both nHL and CLL indications of use was the one that searched diagnostic codes in both the hospital discharge records’ databank and the exemption from copayment registry during all available patients’ observation time before and after cohort entry (i.e., the composite algorithm “EXE*ever* or HDR*ever*”). However, it is important to note that using patients’ observation time before and after cohort entry for event identification may be considered a valid approach only for descriptive cross-sectional studies. For longitudinal comparative studies, this approach has the potential to introduce bias (20).

To the best of our knowledge, this study is the first to provide evidence of the validity of using Italian administrative healthcare data to describe the real-world drug utilization of an infusive antineoplastic drug. Several validation studies have been conducted using administrative/claims databases from other countries, mainly the United States, to identify cancer cases and treatments (21–28). However, comparisons with the present study should be made with caution. For example, a cohort study of patients with nHL in California reported a sensitivity of 57% and a PPV of 94% for identifying patients with CLL using hospital discharge records and medical chart abstraction as the reference standard (21). The same study assessed the validity of hospital discharge records data in identifying chemotherapy users, but only at the drug class level.

Another study reported an overall sensitivity of 78% for Medicare claims data in documenting the correct drugs and schedule for the initial multi-agent chemotherapy regimens in patients with various types of cancer, as compared to data from clinical trials (22). However, this study only measured sensitivity, and the validity of Medicare data in identifying repeated single treatment episodes longitudinally during follow-up was not assessed.

This study had two major strengths: firstly, it was able to link regional administrative data with the database of the Hospital Pharmacy of the University Hospital of Siena, which served as the reference standard for validation. This hospital is one of the largest facilities in the Tuscany region, making this an important resource. Secondly, the study utilized a robust methodology to define, combine, and compare the algorithms tested, adding to the rigor of the study.

As previously demonstrated in multi-national, multi-database studies, the use of simple algorithms based on one single medical concept (such as nHL), one data bank (such as hospital discharge records), and one pre-specified time window for record search ensured transparency and reproducibility of the analysis (29). This approach also allows easy application to any databases. Moreover, the use of administrative healthcare data provides a real-world representation of the utilization of rituximab in clinical practice, which may be more generalizable than data from clinical trials or other selected populations.

The most significant limitation of this study is the generalizability of its findings. It should be noted that caution must be exercised when applying the results of this study to other Italian administrative healthcare databases, other infusive antineoplastics, or other indications for drug use. Other infusive antineoplastics, apart from rituximab, may have a different proportion of administration episodes that are not tracked at the patient-level in Italian administrative data. In fact, drugs given during inpatient care or under off-label/out-of-national-reimbursement criteria are typically tracked at the hospital ward-level, rather than the patient-level. Additionally, the accuracy of identifying the indication of use is expected to depend heavily on the specific condition of interest, its prevalence in the study population, and the granularity of codes used for recording it. Nevertheless, the observation period of this study (2011-2014) coincided with the most recent data set available to us for research purposes from UHS-HPD. Hence, the data lag-time could potentially limit the generalizability of the present study findings. Although we do not anticipate any significant changes in the accuracy of administrative data in the last decade, the growing tendency of Italian hospitals to provide healthcare assistance to oncological patients through outpatient services can have a positive impact on the sensitivity of administrative data in identifying patients treated with rituximab, as well as for the individual episodes of rituximab administration (i.e., drugs given during inpatient care are typically not recorded at the patient-level in Italian administrative data).

In conclusion, this study has generated valuable evidence for future pharmacoepidemiological studies that will use Italian administrative databases to examine the real-world usage of infusive antineoplastics. The results presented here demonstrate that RAD is a highly reliable source of data for identifying patients undergoing treatment with rituximab. Additionally, episodes of rituximab administration were identified with good sensitivity and high positive predictive value. Patients receiving rituximab for nHL were identified with good sensitivity and acceptable PPV. However, the accuracy of identifying those treated for CLL was suboptimal, particularly with regards to PPV. This may be due to the considerably lower prevalence of CLL in the study population compared to nHL. Further validation studies are needed in different geographic areas and with different medications to increase the reliability and quality of real-world pharmacoepidemiological studies on infusive antineoplastics.

## Data availability statement

The data analyzed in this study is subject to the following licenses/restrictions: The datasets presented in this article are not readily available because of the privacy legislation. Requests to access these datasets should be directed to claudia.bartolini@ars.toscana.it.

## Ethics statement

The study protocol was approved by the Ethical Committees of the Local Health Authority “Area Vasta Sud-Est” in July 2016 (protocol identifier: ARSAOUS2016). In accordance with the national legislation and the institutional requirement, written informed consent was not required for inclusion in the present observational study.

## Author contributions

CB, GR, and RG conceived the study. CB, RG and AG developed the study design and the statistical analysis plan. CB and GR performed central data management and analysis. CB, AS, and VM transform local data in common format e run the script for the extraction of the analytical dataset. CB and RG drafted the manuscript with the contribution of AG, GR and MCM. All authors reviewed and approved all steps of the execution of the study including the final version of the manuscript.
