# Supplementary material for: Validity of Italian administrative healthcare data in describing the real-world utilization of infusive antineoplastic drugs: the study case of rituximab use in patients treated at the University Hospital of Siena for onco-haematological indications
Source: Front Oncol. 2023 May 31;13:1059109. doi: 10.3389/fonc.2023.1059109 (PMC10264685; doi:10.3389/fonc.2023.1059109)
Supplement: Supplementary file 1 [file Table_1.docx]

**Supplementary Table 1 – Sensitivity and positive predictive values of the different *simple algorithms* tested for the identification of patients treated with rituximab for nHL and CLL, respectively**

|  | **nHL**  **(n=174)** | | |  | **CCL**  **(n=21)** | |
| --- | --- | --- | --- | --- | --- | --- |
|  | RAD  ∩  HPD-UHS | *Sensitivity (%)*  *[95% CI]** | *PPV (%)*  *[95% CI]** | RAD  ∩  HPD-UHS | *Sensitivity (%)*  *[95% CI]** | *PPV (%)*  *[95% CI]** |
| HDR*ever* | 145 | *83.3*  *[77.7-88.8]* | *65.3*  *[59.1-71.6]* | 12 | *57.1*  *[34.0-78.1]* | *34.3*  *[19.1-52.2]* |
| HDR*pre* | 119 | *68.4*  *[61.8-75.3]* | *69.6*  *[62.7-76.5]* | 5 | *23.8*  *[8.2-47.2]* | *27.7*  *[9.7-53.5]* |
| HDR*pre2* | 116 | *66.6*  *[59.6-73.7]* | *70.7*  *[63.7-77.7]* | 3 | *19.0*  *[5.4-41.9]* | *28.5*  *[8.4-58.1]* |
| HDR*within* | 53 | *30.5*  *[23.6-37.3]* | *71.6*  *[61.3-81.9]* | 0 | *0.0*  *[0-16.1]* | *0.0*  *[0-97.5]* |
| HDR*post* | 113 | *64.9*  *[57.8-72.0]* | *66.5*  *[59.4-73.5]* | 10 | *47.6*  *[25.7-70.2]* | *38.5*  *[20.2-59.4]* |
|  |  |  |  |  |  |  |
| ER*ever* | 21 | *12.1*  *[8.0-18.1]* | *84.0*  *[63.6-95.4]* | 3 | *14.3*  *[9.1-19.5]* | *75.0*  *[19.4-99.4]* |
| ER*pre* | 10 | *5.7*  *[2.9-9.6]* | *90.1*  *[58.9-100.0]* | 2 | *9.5*  *[5.1-13.8]* | *66.6*  *[0.9-99.2]* |
| ER*pre2* | 10 | *5.7*  *[2.9-9.6]* | *90.1*  *[58.9-100.0]* | 2 | *9.5*  *[5.1-13.8]* | *66.6*  *[0.9-99.2]* |
| ER*post* | 14 | *8.0*  *[4.0-12.7]* | *82.4*  *[67.2-96.0]* | 1 | *4.7*  *[1.5-7.9]* | *50.0*  *[1.2-98.8]* |
|  |  |  |  |  |  |  |
| EXE*ever* | 120 | *68.9*  *[62.1-75.8]* | *63.8*  *[56.9-70.6]* | 14 | *66.6*  *[43.0-85.4]* | *41.1*  *[24.6-59.3]* |
| EXE*pre* | 98 | *56.3*  *[48.9-63.7]* | *63.2*  *[55.6-70.7]* | 11 | *52.4*  *[29.7-74.3]* | *36.6*  *[19.9-56.1]* |
| EXE*pre2* | 79 | *49.4*  *[56.8-41.9]* | *62.7*  *[54.6-70.8]* | 4 | *28.6*  *[11.3-52.2]* | *35.3*  *[14.2-61.7]* |
| EXE*post* | 26 | *14.9*  *[9.6-20.2]* | *63.4*  *[48.7-78.1]* | 3 | *14.3*  *[3.0-36.4]* | *50.0*  *[11.8-88.2]* |

PPV=positive predictive value; nHL=non-Hodgkin's lymphoma; CLL= chronic lymphocytic leukemia; HDR= Hospital discharge records; ER= Emergency Room; EXE= Disease-specific exemption from copayement registry

* 95% CI= 95% confidence interval (CI) of the Sensitivity (SENS) and PPV is calculated respectively as *SENS ± Z_α/2_ * sqrt( ( SENS *(1-SENS) )/N)* and *PPV ± Z_α/2_* sqrt( ( PPV *(1-PPV) )/N)*
