# Supplementary material for: Validity of Italian administrative healthcare data in describing the real-world utilization of infusive antineoplastic drugs: the study case of rituximab use in patients treated at the University Hospital of Siena for onco-haematological indications
Source: Front Oncol. 2023 May 31;13:1059109. doi: 10.3389/fonc.2023.1059109 (PMC10264685; doi:10.3389/fonc.2023.1059109)
Supplement: Supplementary file 2 [file Table_2.docx]

**Supplementary Table 2 – Sensitivity and positive predictive values of the different *composite algorithms* tested for the identification of patients treated with rituximab for nHL and CLL, respectively**

|  | **nHL (n=174)** | | | **CCL (n=21)** | | |
| --- | --- | --- | --- | --- | --- | --- |
|  | RAD  ∩  HPD-UHS | *Sensitivity (%)*  *[95% CI]** | *PPV (%)*  *[95% CI]** | RAD  ∩  HPD-UHS | *Sensitivity (%)*  *[95% CI]** | *PPV(%)*  *[95% CI]** |
| (HDR or ER*)pre2* | 116 | *66.6*  *[59.6-73.7]* | *70.7*  *[63.7-77.7]* | 5 | *23.8*  *[7.5-47.1]* | *31.2*  *[10.5-58.9]* |
| (HDR or ER)*pre* | 119 | *68.4*  *[61.5-75.3]* | *69.6*  *[62.7-76.5]* | 6 | *28.6*  *[10.8-52.3]* | *30.0*  *[11.9-54.0]* |
| (HDR or ER)*pre* or HDR*within* | 119 | *68.4*  *[61.5-75.3]* | *69.6*  *[62.7-76.5]* | 6 | *28.6*  *[10.8-52.3]* | *30.0*  *[11.9-54.0]* |
| HDR*pre2* or EXE*pre* | 144 | *82.7*  *[77.1-88.4]* | *65.7*  *[59.5-72.0]* | 12 | *57.1*  *[49.8-64.4]* | *33.3*  *[18.9-50.7]* |
| HDR*pre2* or HDR*within* or EXE*pre* | 144 | *82.7*  *[77.1-88.4]* | *65.7*  *[59.5-72.0]* | 12 | *57.1*  *[33.8-85.4]* | *33.3*  *[18.9-50.7]* |
| (HDR or ER)*ever* | 145 | *83.3*  *[77.7-88.8]* | *65.3*  *[59.0-71.5]* | 14 | *66.6*  *[44.6-84.7]* | *36.8*  *[21.5-54.2]* |
| (HDR or EXE or ER)*pre* or HDR*within* | 146 | *83.9*  *[78.4-89.4]* | *65.7*  *[59.5-72.0]* | 13 | *61.9*  *[37.7-82.1]* | *34.3*  *[20.0-51.1]* |
| HDR*pre* or HDR*within* or EXE*pre* | 146 | *83.9*  *[78.4-89.4]* | *65.7*  *[59.5-72.0]* | 13 | *61.9*  *[54.7-69.1]* | *33.3*  *[18.9-50.7]* |
| (HDR or EXE or ER)*ever* | 160 | *91.9*  *[87.9-95.9]* | *64.7*  *[58.8-70.7]* | 18 | *85.7*  *[63.5-96.9]* | *37.5*  *[23.8-52.5]* |

PPV=positive predictive value; nHL=non-Hodgkin's lymphoma; CLL= chronic lymphocytic leukemia; HDR= Hospital discharge records; ER= Emergency Room; EXE= Disease-specific exemption from copayement registry

* 95% CI= 95% confidence interval (CI) of the Sensitivity (SENS) and PPV is calculated respectively as *SENS ± Z_α/2_ * sqrt( ( SENS *(1-SENS) )/N)* and *PPV ± Z_α/2_* sqrt( ( PPV *(1-PPV) )/N)*
